# Case report: interventional radiology’s potential role for in vitro fertilization post ovarian transposition and pelvic radiation

**DOI:** 10.1186/s40738-019-0056-x

**Published:** 2019-04-02

**Authors:** Benjamin Jang, Aaron Rohr, Paras P. Vakharia, Zachary Collins, Courtney Marsh, Isabella Herrera, Thomas Fahrbach

**Affiliations:** 1 MacNeal Hospital, Internal Medicine, Berwyn, IL USA; 2 Stanford University, Interventional Radiology, Palo Alto, CA USA; 3 University of Kansas, Interventional Radiology, Kansas City, KS USA; 4 University of Kansas, Obstetrics and Gynecology, Kansas City, KS USA; 5 Riverside Community Hospital, Emergency Medicine, Riverside, CA USA; 6 Boulder Community Health, Interventional Radiology, Boulder, CO USA

**Keywords:** Interventional radiology, Ultrasound, Transabdominal, Oocyte, In vitro fertilization

## Abstract

**Introduction:**

Ovarian transposition is a procedure that can help preserve fertility for female patients requiring radiation in the abdominopelvic region. However, the displacement of ovaries from its original anatomic location can make oocyte retrieval challenging.

**Case presentation:**

A 24-year-old nulligravid patient recently diagnosed with colorectal carcinoma [CRC] underwent ovarian transposition prior to radiation. After radiation and chemotherapy, she began in vitro fertilization [IVF] by reproductive endocrinology and infertility physicians. Right ovary demonstrated nonviability due to failed transposition and radiation. Left ovarian oocytes were not able to be harvested due to risk of left kidney puncture via transvaginal ultrasound [TVUS]. Interventional Radiology [IR] was involved and performed a transabdominal ultrasound guided egg retrieval which led to successful IVF.

**Conclusion:**

This case highlights the utility of IR-assisted transabdominal ultrasound approach for oocyte retrieval in patients with history of ovarian transposition.

## Introduction

For fertile female patients diagnosed with abdominopelvic malignancies requiring radiation treatment, preservative measures to reduce infertility may be elected [1]. Ovarian transposition, a surgical maneuver to displace the ovaries away from radiation can be done in women seeking fertility preservation prior to pelvic radiation therapy [2]. This procedure involves the detachment of ovaries and its vasculature from its typical anatomic location and reattaching it caudally away from possible gonadotoxic radiation. Consequently, TVUS oocyte retrieval after ovarian transposition can be challenging due to new pelvic organ orientation [3]. Here we present a case of an IR-assisted transabdominal oocyte retrieval in a patient status post ovarian transposition. This study was deemed exempt from the IRB review panel at our institution and would not require ethical or randomized control trial code.

## Case presentation

**Fig. 1:**
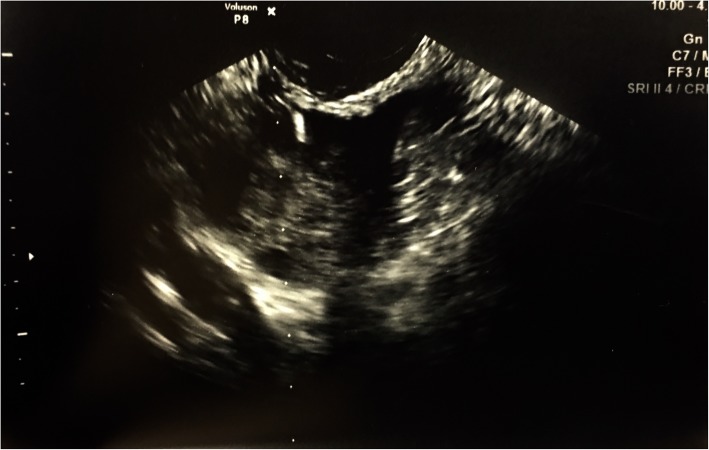
Tip of aspiration needle inside a follicle under ultrasound

**Fig. 2:**
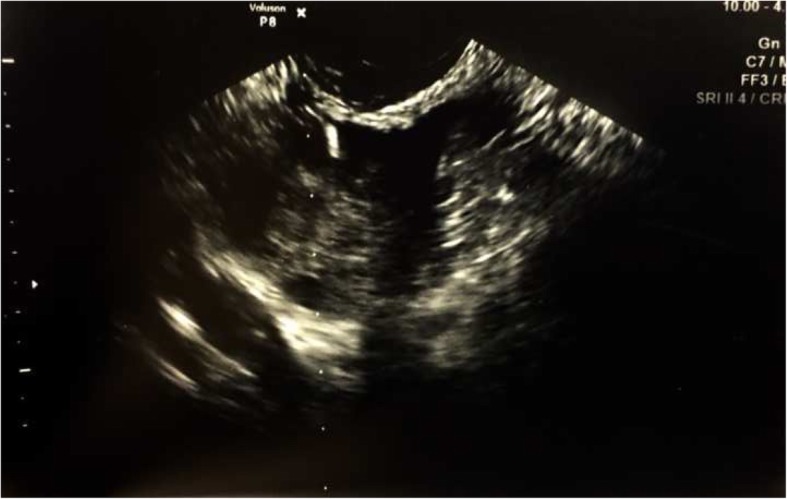
Ultrasound of collapsed follicle after oocyte aspiration

A 24-year-old nulligravid patient diagnosed with CRC required radiation and chemotherapy. To reduce radiation exposure, the patient had her ovaries detached from the fallopian tubes and transposed to her abdominal oblique muscles. Although radiation exposure was limited, the ovaries were still exposed to chemotherapy. After chemoradiation therapy, she began IVF with reproductive endocrinology. Initially, only the right ovary was visualized by TVUS and two eggs were retrieved. However, the IVF procedure was unsuccessful. Unfortunately, the right ovary was later found to be in failure – it did not remain in its new post-surgical anatomic location and subsequently was exposed to radiation treatments. Additionally, the left ovary was found to be displaced from the oblique muscles and was nestled near the left kidney. Due to the new location of left ovary, egg retrieval using transvaginal approach posed a risk of puncturing the left kidney. Thus, IR prepared to perform a transabdominal ultrasound guided oocyte collection. Abdominal ultrasound examination was performed to provide a pre-procedural approach. After visualization of ovarian follicles, the skin and subcutaneous tissue were anesthetized with lidocaine. A 3 mm skin incision was made with a scalpel and a 16-gauge aspiration needle [Vitrolife single lumen; Goteborg, Sweden] was advanced under ultrasound guidance. The needle tip was placed within the follicle and was suctioned until the follicular wall collapsed [Figs. 1 and 2]. Suction was then released before the needle was withdrawn, repositioned, and re-advanced to aspirate more follicles while minimizing the abdominal punctures. From the twelve eggs retrieved, ten were mature, seven were fertilized, three embryos were formed, and one of them led to a healthy boy.

## Discussion

This case illustrates the importance of multidisciplinary teamwork to prepare ovaries prior to radiation for a successful IVF. To avoid ovarian radiation, patients can have their ovaries relocated above and out of their true pelvis. This ovarian transposition procedure can lead to 65–90% preservation of ovarian function based on menopausal symptoms and hormone levels of estradiol, follicle stimulating hormone, and luteinizing hormone. This viability was followed up on average 31.4 months [3]. For premenopausal patients requiring pelvic radiotherapy, discussion of ovarian transposition can be beneficial. However, abdominopelvic surgery can lead to challenging transvaginal oocyte retrieval due to altered pelvic organ anatomy [4]. For patients that have these variations, transabdominal oocyte retrieval may be used for ovarian access and avoid laparoscopic surgery [5]. There has been multiple studies of successful transabdominal oocyte retrieval and it can be a safe and efficacious alternative to the transvaginal approach [5–8]. Reproductive endocrinology and infertility physicians are generally trained and regularly perform transabdominal oocyte retrievals. However, this case is unique in demonstrating the ultrasound guided transabdominal oocyte retrieval done by IR. This also allows the patients and their reproductive endocrinology and infertility physicians to be aware of IR’s potential role of assistance in these type of cases.

## Abbreviations

CRC: Colorectal cancer
IR: Interventional radiology
IVF: In vitro fertilization
TVUS: Transvaginal ultrasound
